# Optimizing Resource Management with Organic Fertilizer and Microbial Inoculants to Enhance Soil Quality, Microbial Diversity, and Crop Productivity in Newly Cultivated Land

**DOI:** 10.3390/plants14193032

**Published:** 2025-09-30

**Authors:** Yuling Dai, Xiaoxiao Wu, Shuo Li, Yan Li, Lei Wang, Yu Hu, Kangmeng Liu, Zhenguo Yang, Lianfeng Cai, Kuifeng Xu, Meili Cui, Xuening Xu, Yuehui Jia, Dan Wei, Jianli Ding

**Affiliations:** 1 Institute of Plant Nutrition, Resources and Environment, Beijing Academy of Agriculture and Forestry Sciences, Beijing 100097, China; 19960781526@163.com (Y.D.); wuxx_gsau@163.com (X.W.); yzswanglei@163.com (L.W.); huyu0805@126.com (Y.H.); lkm0814@163.com (K.L.); 18330382950@163.com (Z.Y.); clfcx13027878049@163.com (L.C.); 19164907562@163.com (K.X.); 18713836012@163.com (M.C.); xvmm1316@163.com (X.X.); 2 College of Resources and Environment, Northeast Agricultural University, Harbin 150030, China; 3 Heilongjiang Academy of Black Soil Protection and Utilization, Harbin 150086, China; li.yan622@163.com; 4 College of Plant Science and Technology, Beijing University of Agriculture, Beijing 102206, China; yhjia@bua.edu.cn

**Keywords:** newly cultivated land, soil quality, soil bacterial community, crop productivity

## Abstract

In response to China’s drive to bring newly cultivated land into production, this study evaluated how combined organic fertilizer and microbial inoculants affect soil quality, bacterial community structure, and maize yield. Four treatments were evaluated: FC (chemical fertilizer only), T50 (50% organic fertilizer + 50% chemical fertilizer), T50M (T50 plus microbial inoculant), and CK (no fertilizer). T50M significantly increased yield compared to FC and CK (*p* < 0.05), achieving the highest yield of 6995.73 kg ha^−1^. This was 20.09% greater than FC. Community composition analyses showed that soil in newly cultivated land was dominated by Blastocatellia, Vicinamibacteria, and Alphaproteobacteria, together accounting for over 35.7% of total bacterial abundance. Redundancy analysis at the class level explained 55.7% of variance; soil organic matter (SOM) and available potassium positively correlated with Alphaproteobacteria and Bacteroidia, while available phosphorus and nitrate nitrogen aligned with Actinobacteria and Bacilli. Path analysis indicated that SOM and total nitrogen were the strongest positive drivers of yield. Actinobacteria and Acidobacteriae also showed direct positive effects, whereas Verrucomicrobiae had a negative effect. These results demonstrate that integrated organic–microbial amendments can enhance soil fertility and alter microbial diversity toward taxa that can improve maize productivity.

## 1. Introduction

China has a cultivated land area of 12.8 million ha, although the per capita cultivated land in China is only one-third of the global average [1,2]. This creates a particularly pronounced conflict between land and population resources. With ongoing industrialization and urbanization, agricultural land is being transferred to urban construction and other uses. The North China region, an important grain production area in the country [1,3], is also one of the regions undergoing rapid urbanization [4], which exacerbates this issue. Because of China’s limited cultivated land area, it is crucial to effectively use and develop land resources through the ‘land occupation and compensation balance’ policy [5,6]. However, newly developed farmland often suffers from poor soil structure, low fertility levels, and insufficient crop productivity [7,8,9], which creates significant challenges for agricultural production [10,11]. Therefore, the implementation of optimized soil management practices is vital to improve the soil quality of new farmland, to ensure food security, and to promote sustainable agricultural development.

Chemical nitrogen fertilizers are an important measure to enhance soil productivity; however, they can lead to soil compaction and environmental degradation [12,13,14]. Organic fertilizer application, as an environmentally sensitive soil management measure, can boost soil quality, strengthen microbial activity, and elevate crop yields [15,16,17,18]. Previous studies have indicated that the rich organic matter in organic fertilizers can improve the physical and chemical properties of soil [19], such as increasing soil aggregate stability and nutrient supply capacity, and enhancing water retention capacity [20,21]. Also, it can promote the diversity and activity of soil bacterial communities by providing ample carbon sources and nutrients [22,23]. These modifications boost plant growth, strengthen crop stress resistance, and elevate yields [24]. However, the specific effects of organic fertilizer application are influenced by factors such as soil type and methods of application [25,26]. Therefore, it is essential to study how organic fertilizers affect soil health indicators in newly cultivated land and if they can enhance essential ecosystem services.

In recent years, with the increasing demand for sustainable agricultural development, microbial inoculants have gradually attracted attention as emerging soil conditioners. Application of microbial inoculants can significantly alter microbial community structure and soil enzyme activity in soil [27,28,29,30]. Studies have shown that the diversity of soil microorganisms increases after the addition of microbial inoculants, with a significant rise in the abundance of functional microorganisms such as nitrogen-fixing bacteria and phosphorus-solubilizing bacteria [31]. These changes help to improve natural ecological functions and enhance self-regulating capacity [32], thus improving soil fertility and structure. Research has indicated [33] that fields treated with microbial inoculants show a 15–30% increase in soil organic matter (SOM) content compared to control groups. However, recent studies have mainly focused on the separate application of organic fertilizers or microbial inoculants. The combined effects of both on soil bacterial communities and physicochemical properties, as well as their contributions to crop yield, still remain unclear.

This study seeks to (1) examine the effects of fertilizer regimes on soil nutrient levels, soil microbial community composition, and variations in dominant microorganism groups in newly cultivated land; (2) clarify the response relationship between soil characteristics, bacterial community, and crop yield when organic fertilizer is used with microbial inoculants in newly cultivated land; and (3) identify the main factors influencing productivity and microbial community structure in newly cultivated land. The aim is to establish a theoretical foundation for the rapid improvement of soil quality in newly cultivated land.

## 2. Results

### 2.1. Soil Properties

**Figure 1:**
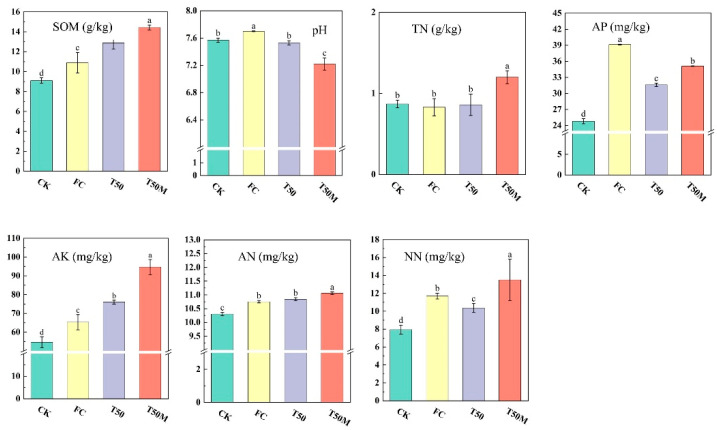
Effects of different treatments on physical and chemical properties of newly added cultivated land. TN: total nitrogen, SOM: soil organic matter, pH: acidity, AP: available phosphorus, AK: available potassium, AN: ammonium nitrogen, NN: nitrate nitrogen; CK: no fertilizer, FC: chemical fertilizer, T50: 50% organic fertilizer +50% chemical fertilizer, T50M: 50% organic fertilizer +50% chemical fertilizer + microbial inoculants. Lowercase letters were used to indicate a significant difference between groups (α = 0.05).

SOM treated with FC, T50, and T50M reached 10.89, 12.87, and 14.42 g/kg, respectively, all of which were significantly higher than in the untreated control (CK) (*p* < 0.05). The T50M treatment resulted in the largest increase in SOM, raising it by 32.42% compared with FC. Ammonium nitrogen (AN), nitrate nitrogen (NN), total nitrogen (TN), and available potassium (AK) also peaked under T50M, measuring 11.06 mg/kg, 13.49 mg/kg, 1.20 g/kg, and 94.67 mg/kg, respectively, which corresponds to increases of 2.88%, 15.40%, 44.58%, and 44.90% compared to FC. Regarding available phosphorus (AP), FC treatment had the highest content (39.15 mg/kg), while T50M had an AP content of 35.10 mg/kg. Although the AP content in T50M was lower than in the FC treatment, it still remained at a relatively high level. In addition, T50M treatment significantly reduced soil pH value, which decreased by approximately 0.48 units compared with FC, demonstrating its potential role as a soil improver (Figure 1).

### 2.2. Soil Bacterial Community Structure and Diversity

#### 2.2.1. Analysis of Soil Bacterial Community α-Diversity Index, and OTU Composition

**Figure 2:**
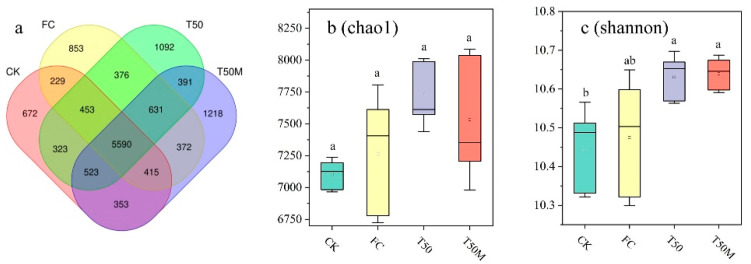
Composition of soil bacterial community OTUs (**a**), Chao 1 index (**b**), and Shannon index (**c**). Lowercase letters were used to indicate a significant difference between groups (α = 0.05).

A total of 200,136 valid sequences were obtained from 16S rRNA high-throughput sequencing. Clustering analysis based on a 97% similarity threshold resulted in 13,491 OTUs, representing 47 phyla, 131 classes, 329 orders, 492 families, 906 genera, and 892 species. Among the four treatments, a total of 5590 OTUs were identified. The T50M treatment had 1218 unique bacterial OTUs, while T50 had 1092 unique OTUs, FC had 853 unique OTUs, and CK had 672 unique OTUs (Figure 2a). Representative Chao 1 and Shannon indices were selected to reflect bacterial community richness and diversity, as shown in Figure 2b. The Chao 1 index for T50 was 7724.69, which was 8.77% and 6.31% higher than CK and FC, respectively. Chao 1 index for T50M was 7531.84, representing increases of 8.06% and 3.66 compared to CK and FC, respectively. This reflects soil microbial richness resulting from T50 and T50M. As shown in Figure 2c, the Shannon indices for the T50 and T50M treatments were significantly higher than those of CK and FC, demonstrating that both organic fertilizers and microbial inoculants contribute to enhancing the diversity of soil bacteria.

#### 2.2.2. Structural Composition of Soil Bacterial Communities

**Figure 3:**
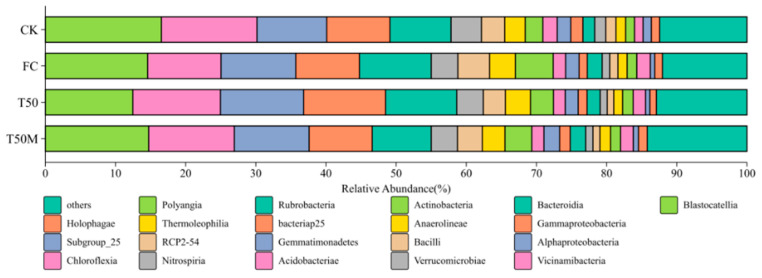
Composition of soil bacterial community.

The soil bacterial community composition under each treatment is presented in Figure 3. Illumina MiSeq sequencing detected 131 bacterial classes. Across all treatments, the combined relative abundance of Blastocatellia, Vicinamibacteria and Alphaproteobacteria exceeded 35.70%, indicating their dominance. Compared with the CK, the abundance of Blastocatellia declined by 11.75% in FC, 24.66% in T50 and 11.59% in T50M. Similarly, Acidobacteriae decreased by 14.22%, 18.03% and 16.77% under FC, T50 and T50M, respectively. In contrast, Alphaproteobacteria increased in all fertilized treatments, by 6.70% in FC, 18.13% in T50 and 6.72% in T50M. The most pronounced change was observed in Actinobacteria, whose abundance rose by 113.06% under FC, 28.01% under T50 and 51.68% under T50M.

#### 2.2.3. Analysis of Bacterial Community Structure Differences and Correlation Analysis

**Figure 4:**
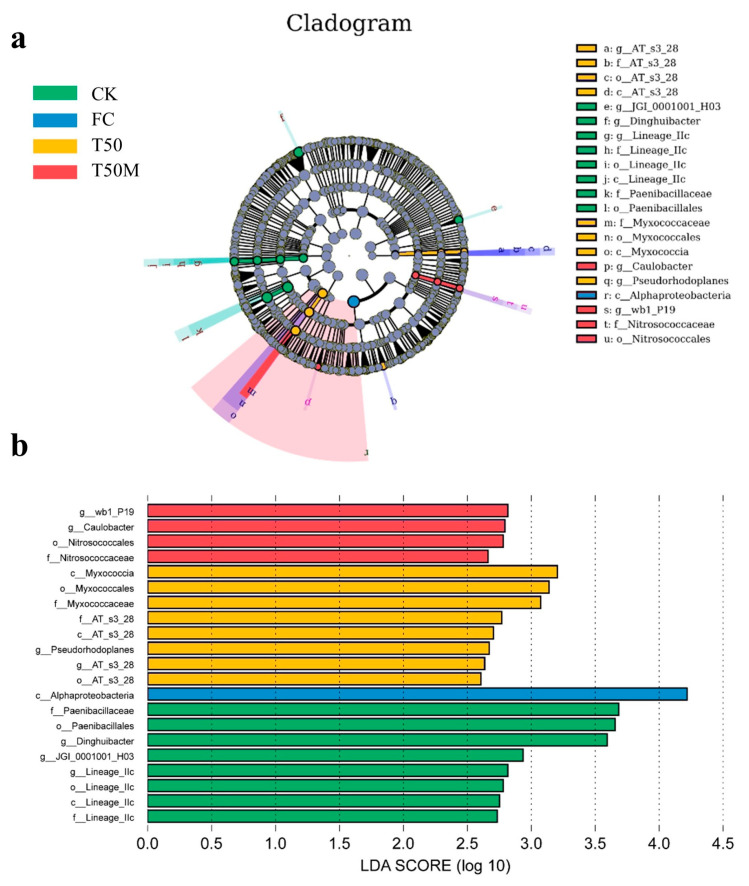
Evolutionary branching diagram of soil bacterial communities (**a**), distribution of LDA values (**b**).

In CK, the indicator bacteria in the soil were mainly from the phylum Bacilli (including the family Paenibacillaceae and the order Paenibacillales) and the genus *Dinghuibacter*. In the FC treatment, fewer bacterial taxa were enriched, with only members of the order Alphaproteobacteria showing significant presence. In the T50 treatment, dominant bacterial taxa included the family Myxococcaceae, belonging to the order Myxococcales and class Myxococcia. For the T50M treatment, all indicator taxa had LDA scores below 3. The indicator bacteria were mainly from class Gammaproteobacteria (including the genus *wb1_P19*, the order Nitrosococcales and the family Nitrosococcaceae) and the genus *Caulobacter* (Figure 4a,b).

**Figure 5:**
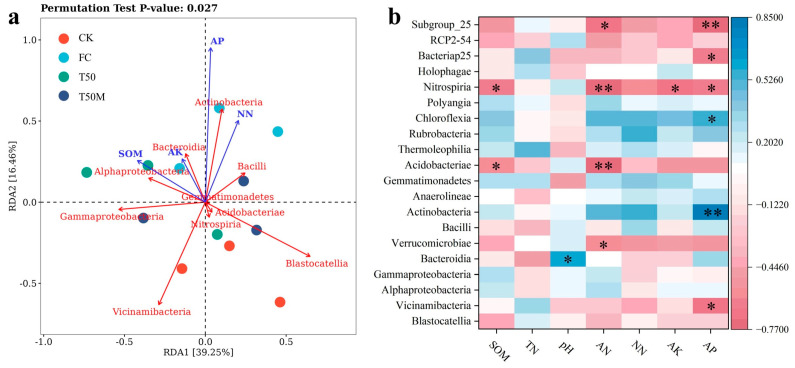
Redundancy analysis (**a**) and correlation analysis (**b**) of soil physicochemical properties and bacterial communities. * represent *p* < 0.05, ** represent *p* < 0.01.

Redundancy analysis at the class level explained 55.71% of total variance (RDA1 = 39.25%, RDA2 = 16.46%; Figure 5a). SOM and AK correlated positively with Alphaproteobacteria and Bacteroidia and inversely with Nitrospiria, Acidobacteriae, and Blastocystidea. In contrast, AP and NN aligned positively with Alphaproteobacteria, Bacteroidia, Actinobacteria, and Bacilli, while showing negative associations with Vicinamibacteria and Gammaproteobacteria.

Spearman rank correlations further clarified how soil properties shape bacterial communities (Figure 5b). SOM was negatively associated with Acidobacteriae and Nitrospiria (*p* < 0.05). pH correlated positively with Bacteroidia (*p* < 0.05). AN showed negative correlations with Verrucomicrobiae and Subgroup 25 (*p* < 0.05), and even stronger negative associations with Acidobacteriae and Nitrospiria (*p* < 0.01). AK correlated negatively with Nitrospiria (*p* < 0.05). AP was positively linked to Actinobacteria and Chloroflexia and negatively to Subgroup_25, Bacteriap25, Nitrospiria, and Vicinamibacteria (*p* < 0.05). Neither NN nor TN exhibited significant correlations with any bacterial group. These patterns indicate that nutrient availability is a major determinant of microbial community composition.

### 2.3. Maize Morphology and Yield

**Figure 6:**
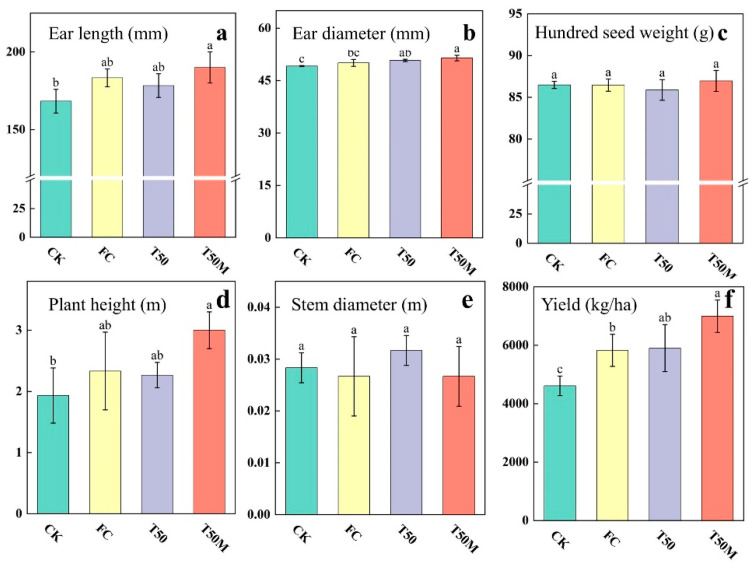
Maize morphology (**a**–**e**) and yield (**f**) in newly cultivated land under different treatments. Lowercase letters were used to indicate a significant difference between groups (α = 0.05).

The effects of different treatments on maize morphology and yield are shown in Figure 6. Both T50 and T50M showed positive effects relative to CK and FC. Compared with FC, the T50M treatment significantly increased ear length by 3.64%, ear diameter by 2.80%, and plant height by 28.57% (*p* < 0.05). Under T50, ear diameter and stem diameter increased by 1.42%, and 18.75%, respectively, compared to FC. Across all treatments, maize yield ranged from 4612.37 to 6995.73 kg ha^−1^, and each fertilizer regime produced a significantly higher yield than CK (*p* < 0.05). The highest yield was observed under T50M, with a significant increase of 20.09% compared to FC (*p* < 0.05). Similarly, T50 yielded 1.34% more than FC.

### 2.4. Direct and Indirect Effects of Soil Properties and Bacterial Communities on Yield (Path Analysis)

**Figure 7:**
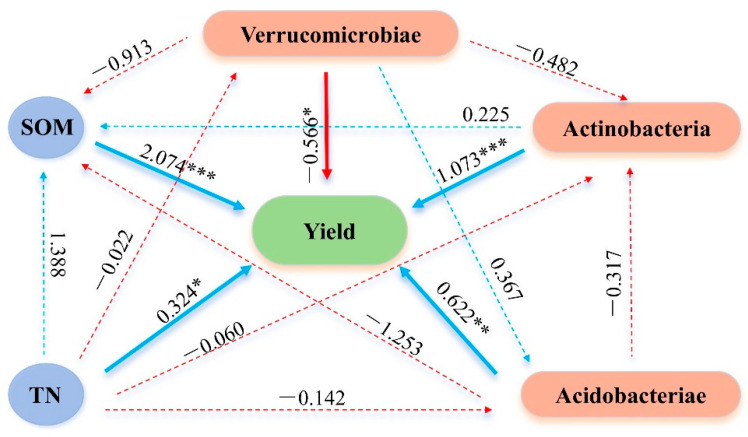
Path analysis between selected soil properties, microbial community, and maize yield. *, ** and *** represent *p* < 0.05, *p* < 0.01 and *p* < 0.001, respectively.

By using a stepwise regression model, it was determined that SOM, TN, Actinobacteria, Verrucomicrobiae, and Acidobacteriae made the largest contribution to maize yield. Therefore, these five independent variables were selected for path analysis (R^2^ = 0.989, *p* = 0.001). Path analysis revealed that SOM (path coefficient = 2.0744, *p* < 0.001) and TN (0.3244, *p* < 0.05) were the strongest positive soil predictors of yield. Among microbial factors, Actinobacteria and Acidobacteriae each showed highly significant positive effects (*p* < 0.001 or *p* < 0.01), whereas Verrucomicrobiae had a significant negative effect (*p* < 0.05). Overall, SOM and Actinobacteria emerged as the principal drivers of productivity, while TN and Acidobacteriae influenced yield primarily by their influence on SOM (Figure 7).

## 3. Discussion

### 3.1. Responses of Different Treatments on the Soil Properties of Newly Cultivated Land

Organic fertilizers increase organic matter content in soil, promote the binding of soil particles, improve soil structure, and form soil aggregates with a good water retention capacity [34,35,36]. The loose structure can enhance nutrient retention and supply capacity; aggregates rich in organic matter can retain more nutrients (such as NN, AK) by adsorption, reducing nutrient leaching caused by rainwater erosion or irrigation. In addition, the microenvironment inside soil aggregates can provide a stable habitat for microorganisms, promote the decomposition of organic matter and nutrient transformation, and gradually release available nutrients for crop use, achieving ‘long-term nutrient supply’ [37,38]. The results of the study also confirm this: in the T50 and T50M treatments, the nutrient contents of NN, TN, AN, and AK were all much higher than those in the chemical fertilizer-applied treatment group (FC) (Figure 1).

Nitrogen in soil plays a crucial role in crop yield [39,40], and TN is one of the most important indicators in soil. Our research indicated that the application of organic fertilizer combined with microbial inoculants significantly increased TN content in soil by 44.57% compared to FC. This enhancement was attributed to the high clay-to-sand ratio in the organic fertilizer and microbial inoculants, which directly increase soil clay content, facilitate more nitrogen retention and elevate nitrogen levels in newly cultivated soil [41,42,43,44].

A modest but consistent decline in soil pH was observed in T50M, amounting to a 6.2% reduction relative to FC (Figure 1). Two linked processes likely explain this change. First, the substantial increase in SOM following the application of organic fertilizer provides substrate for microbial decomposition, which produces organic acids and CO_2_. The latter dissolves to form carbonic acid, and together these products raise hydrogen ion concentration in the soil solution. Second, the introduced Bacillus-based inoculant likely amplified these transformations: the inoculated strains have a high metabolic activity and can secrete organic acids directly and accelerate the mineralization of organic substrates, thereby increasing the production of acidic intermediates. Newly cultivated soils that tend to be alkaline benefit from this mild acidification because it promotes desorption and solubilization of otherwise fixed nutrients (notably phosphorus and some potassium forms). This increases their plant-available pools and supports crop uptake [33,45]. This mechanism is consistent with the stronger nutrient indicators and the superior yield observed under T50M in our study.

Both T50 and T50M increased nutrient concentrations in the newly cultivated soil and improved indicators of fertility more effectively than FC. These improvements, such as increased SOM accumulation, enhanced available nutrients, and improved nutrient absorption by maize, are consistent with previous reports, showing that organic amendments, with or without microbial inoculation, can more rapidly rebuild soil function on marginal or newly developed land [46]. Taken together, our results suggest that integrating organic inputs with targeted microbial amendments offers a practical strategy for restoring soil fertility and optimizing nutrient availability in newly cultivated fields.

### 3.2. Effects of Different Treatments on Soil Bacterial Community in Newly Cultivated Land

Microbial inoculants have become common in modern agriculture [47], and long-term reclaimed and established cropland soils often harbor abundant Actinobacteria and Chloroflexi, which can comprise a substantial fraction of the bacterial consortia [48,49]. In our newly cultivated plots, these taxa were not initially dominant; however, the combined application of organic fertilizer and microbial inoculants increased their relative abundances by 51.68% and 45.32%, respectively. This shift demonstrates that targeted amendments can accelerate the development of a more functionally diverse microbial community, thereby aiding soil quality restoration in newly developed farmland.

Newly developed soils typically suffer from low organic matter and limited microbial functionality [50]. Adding organic fertilizer enriches the soil with carbon, nitrogen, and essential micronutrients, supporting the proliferation of a wider range of microorganisms [51,52]. As a result, overall microbial diversity and abundance rise, strengthening ecosystem stability and resilience to environmental stress [25,53,54]. In our study, Alphaproteobacteria increased by 18.13% under organic fertilization, which is consistent with observations in other carbon-enriched soils [55].

Supplementing with microbial inoculants further promoted beneficial taxa: the abundance of Gemmatimonadetes rose by 14.84%, likely reflecting the high species richness within commercial inoculant formulations that bolster soil bacterial populations and nutrient-cycling processes [56,57]. Together, organic and microbial amendments enhanced microbial diversity and activity and also improved soil physicochemical properties and accelerated nutrient turnover. The superior performance of T50M over T50 can be attributed to the synergy between organic fertilizer and microbial inoculants: organic fertilizer provides sufficient carbon/nutrient substrates and stable microhabitats for the colonization and proliferation of inoculated Bacillus strains, which in turn enhance nutrient cycling (e.g., nitrogen transformation, phosphorus/potassium activation) and enrich beneficial taxa (e.g., Actinobacteria, Gammaproteobacteria), ultimately improving soil fertility and maize yield [33]. Meanwhile, the greater number of distinct OTUs observed under T50M may reflect either establishment of introduced strains or stimulation of rare native taxa [58]; current 16S data do not allow unambiguous attribution. Resolving this requires sequencing the inoculant and applying targeted approaches such as strain-specific qPCR, source-tracking analyses, or comparative metagenomics in future studies.

### 3.3. Relationship Between Soil Properties, Bacterial Community Structure, and Crop Yield

In this study, the path analysis was employed to identify and quantify the principal soil properties and bacterial communities driving maize yield. The results indicated that SOM (path coefficient = 2.0744, *p* < 0.001) and TN (coefficient = 0.3244, *p* < 0.05) were the primary soil properties drivers of yield, corroborating extensive evidence that SOM and TN underpin crop productivity [59,60].

Among microbial predictors, Actinobacteria (*p* < 0.001) and Acidobacteriae (*p* < 0.01) exerted significant positive effects on maize yield, whereas Verrucomicrobiae had a negative effect (*p* < 0.05). These statistical associations are consistent with functional roles reported for these groups. Actinobacteria include taxa with strong capacities for degrading complex organic matter, producing extracellular enzymes and secondary metabolites, mobilizing phosphorus, and in some cases acting as endophytes that can benefit plant health; such traits plausibly enhance nutrient release and crop performance [61,62]. Acidobacteriae have been reported to participate in multiple element cycles, to contribute to extracellular polysaccharide production which can influence aggregation and water retention capacity, and in some cases to produce compounds that influence plant growth [63]. The negative association of Verrucomicrobiae, often considered oligotrophic, likely signals that when such taxa are relatively abundant, soils are comparatively low in labile C and available nutrients, conditions that are less favorable for high crop productivity [64]. The superior performance of T50M compared with T50 is best interpreted as a synergistic interaction between the organic substrate and the microbial inoculant. Organic fertilizer supplies labile carbon and nutrients and creates microsites that facilitate microbial colonization; the Bacillus-based inoculant can then more readily establish, express degradative and nutrient-mobilizing functions, and stimulate beneficial indigenous taxa, thereby accelerating nutrient mineralization and improving plant uptake. Furthermore, we are also aware that fungi, archaea and protists play a crucial role in soil processes, and fungal communities are particularly important in organic matter decomposition and plant-pathogen dynamics [65]. In future work, it will complement fungal community analysis and metagenomic methodology to provide a broader and multi-disciplinary perspective.

## 4. Materials and Methods

### 4.1. Study Site

The experiment was conducted at the Scientific Research Base of Heishansi Technology Courtyard, Miyun District, Beijing (E 116°47′23″, N 40°29′35″). The experiment started in May 2023. The climate has an annual average temperature of 20.3 °C, annual average precipitation of 551.6 mm, an average of 1702 sunshine hours, and an average frost-free period of 204 days. Prior to the experiment, the soil had the following basic physicochemical properties: pH 7.54, SOM 9.01 g/kg, TN 1.0 g/kg, AP 16.31 mg/kg, and AK 83.44 mg/kg.

### 4.2. Experimental Design and Materials

**Figure 8:**
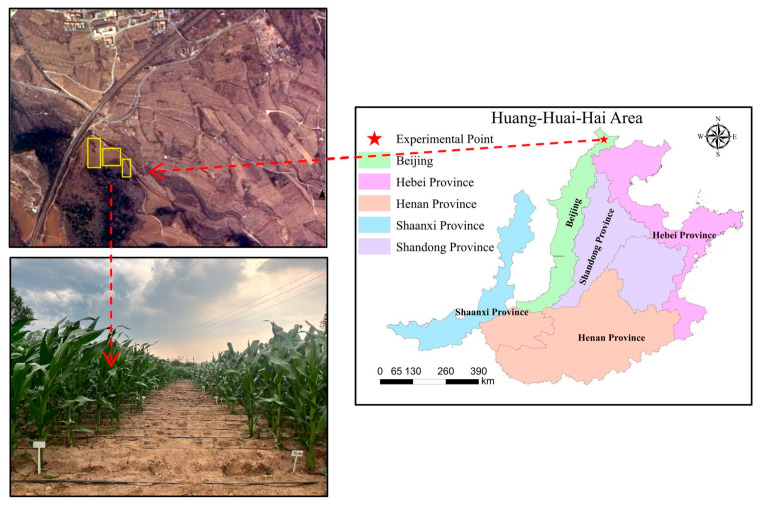
Experimental site distribution map.

This experiment employed a randomized block design, consisting of four experimental treatments, each replicated three times, resulting in 12 plots in total (Figure 8). The treatments were as follows: CK: no fertilizer; FC: 100% chemical fertilizer; T50: 50% organic fertilizer + 50% chemical fertilizer; T50M: 50% organic fertilizer + 50% chemical fertilizer + microbial inoculants. Each plot was 20 m^2^ in size. The nitrogen application rate for all treatments was 240 kg/ha. A single application of organic fertilizer was made as the base dressing, while chemical nitrogen fertilizer was applied in two parts: basal and top dressing, at a ratio of 2:1. A single application of phosphorus and potassium fertilizer was also used as a base dressing. The microbial inoculant in powder form was initially diluted with water at a ratio of 600–800 times and subsequently applied via flushing during the same period as the base fertilizer. The mineral fertilizer employed in this research contained urea (N ≥ 46%, Liaoning Sequoia tree agricultural Technology Co., Ltd., Liaoning, China), calcium superphosphate (P_2_O_5_ ≥ 12%, Hubei Fengle Ecological Fertilizer Co., Ltd., Hubei, China) and potassium chloride (K_2_O ≥ 60%, Cargill, MN, USA). The organic fertilizer was a compost product prepared from straw and cattle manure, supplied by Beijing Woshengjie Planting Soil Co., Ltd. (Beijing, China). Its measured nutrient composition was total N 3.37%, total P_2_O_5_ 2.27%, total K_2_O 21.56% and organic matter 45.50%. The microbial inoculant was a Bacillus-based consortium containing *Bacillus thuringiensis*, *Bacillus licheniformis* and *Bacillus subtilis*, with a guaranteed composite viable count of at least 2.0 × 10^9^ CFU per product unit (supplied by Henanwopu Feng Fertilizer Co., Ltd., Henan, China). The specific fertilizer details are provided in Table 1.

Maize was sown on 5 June 2023, and harvested on 7 October 2023. Protective rows were established around each plot to prevent cross-fertilization. To account for marginal effects, protective areas were set up around the treatments, and other field cultivation measures complied with local management guidelines. The maize variety used for the experiment was Dongdan 1331 (Liaoning Dongya Seed Industry Co., Ltd., Shenyang, China), and newly cultivated soil served as the test soil for the field experiment. The planting distance between maize plants was 0.30 m, and the row spacing was 0.60 m.

### 4.3. Sample Collection and Processing

At a soil depth of 0–20 cm, five samples were obtained following an S-shaped sampling pattern. Stones, roots, and other debris were manually removed prior to homogenizing the collected soil. The homogenized soil was subsequently split into two subsamples. One portion was sealed in sterile polyethylene bags, quickly transported to the laboratory in an insulated cooler, and this fresh subsample was later used for direct extraction of total microbial DNA for high-throughput sequencing. The other subsample was air-dried, sieved (2 mm), and used for physicochemical characterization. Employing a potentiometric approach (DDS-BPA pH meter, Shanghai LeiCi), soil pH was determined at a water-to-soil ratio of 2.5:1 (*w*/*v*). TN was determined by the semi-micro Kjeldahl method, AN by potassium chloride extraction and UV spectrophotometry, and NN by calcium chloride extraction and colorimetry. AP was determined using the sodium bicarbonate extraction-molybdenum-antimony colorimetric method, AK by flame photometry, and SOM by the K_2_Cr_2_O_7_-concentrated H_2_SO_4_ external heating method. The methods for soil parameter measurement followed the protocols described in Methods of Soil Agrochemical Analysis and Soil Agrochemical Analysis [66]. When the maize plants were mature, a five-point sampling method was adopted in each treatment plot, with three uniformly grown, disease- and pest-free maize plants selected at each point, totaling 15 plants. These were used for the determination of the following morphological indicators: plant height, stem diameter, ear length, ear diameter, and 100-grain weight. For the determination of maize yield, a plot harvest method was used, and the yield per hectare was calculated based on the actual planting area of the plot.

### 4.4. Microbial Measurement and Analysis Methods

Genomic DNA was isolated from soil specimens employing the E.Z.N.A. Soil DNA Kit (Omega Bio-tek, Norcross, GA, USA). Amplification of the bacterial *16S rRNA* gene V3-V4 hypervariable region utilized universal primer pairs 338F (5′-ACTCCTACGGGAGGCAGCAG-3′) and 806R (5′-GGACTACHVGGGTWTCTAAT-3′). Unique 8 bp barcode sequences were incorporated into the primer set to enable sample multiplexing. Amplification success was verified by electrophoresing PCR amplicons on a 1% agarose gel. Post-amplification purification was conducted with the Agencourt AMPure XP purification system (Beckman Coulter, Brea, CA, USA). Sequencing libraries were prepared with the NEB Next Ultra II DNA Library Prep Kit (New England Biolabs, Ipswich, MA, USA), followed by a secondary purification step using the Agencourt AMPure XP kit. Final paired-end sequencing was executed on an Illumina MiSeq platform (Illumina, San Diego, CA, USA).

### 4.5. Statistical Analysis and Data Processing

Initial data organization and calculations were carried out in Microsoft Excel 2019. Visualization of soil physicochemical parameters utilized Origin 2022. Statistical analyses, including significance testing (ANOVA/Tukey’s HSD) and Pearson correlation analysis were executed in IBM SPSS Statistics 27.0.1. Alpha diversity indices (Chao1, Shannon) and OTU clustering were computed through QIIME v1.8.0. Microbial community composition constrained by environmental variables was assessed via redundancy analysis (RDA) implemented in Canoco 5.0.

To further investigate the relationships between soil properties and bacterial community composition and their effects on crop yield, the direct and indirect effects of candidate factors on yield was quantified using path analysis. Path analysis was implemented in the Data Processing System (DPS) v 9.01 software by constructing stepwise regression models and a path model [67,68]. The workflow was as follows: (1) Spearman correlation analysis was used to obtain the correlation coefficients between each candidate predictor and yield, and among predictors themselves. (2) Stepwise regression was applied to remove variables with small effects or those causing multicollinearity, thereby identifying the dominant predictor set. (3) The regression coefficients of these dominant factors were standardized to obtain the direct path coefficients; indirect path coefficients were then calculated from products of direct coefficients along indirect paths, allowing estimation of each factor’s relative contribution to yield. The path model may be expressed as:

(1) $$Y_{b}=\sum_{a\neq b}(R_{a,b}X_{a})+w_{a}$$

(2) $$R_{a,b}=\frac{\left(\sum_{j=1}^{i}X_{a,j}Y_{b,j}-\frac{1}{i}\sum_{j=1}^{i}X_{a,j}\sum_{j=1}^{i}Y_{b,j}\right)}{\sum_{j=1}^{i}{X_{a,j}}^{2}-{\frac{1}{n}(\sum_{j=1}^{i}X_{a,j})}^{2}}$$

where Y_b_ is the dependent variable (yield), Xa are the explanatory variables, R_a,b_ denotes the path (regression) coefficient quantifying the direct effect of X_a_ on Y_b_, and w_b_ is the random error term for Y_b_. X_a,j_ and Y_b,j_ are the observed values of X_a_ and Y_b_ for sample j, and n is the total sample size.

All steps, including correlation screening, stepwise variable selection, standardization, path coefficient estimation, and calculation of indirect and total effects, were performed in DPS, and model fit and coefficient significance were evaluated before interpreting factor contributions to yield.

## 5. Conclusions

Our results demonstrate that combined organic fertilizer and microbial inoculant (T50M) applied to newly cultivated soils delivers the highest short-term maize yield and reorganizes microbial communities in ways that reinforce soil function. RDA showed that SOM and AK structure bacterial assemblages, while AP and NN recruit key copiotrophic groups. Path analysis further pinpointed SOM, TN, and copiotrophic taxa (Actinobacteria, Acidobacteriae) as principal positive drivers of yield, whereas oligotrophic Verrucomicrobiae negatively impacted productivity. By directly improving soil nutrient status while simultaneously fostering beneficial microbial populations, this approach provides a powerful means to develop fertile, biologically active soils in newly cultivated farmland. Tailoring fertilizer treatments to foster phosphorus- and carbon-cycling bacteria, rather than relying solely on mineral nitrogen, will optimize both soil health and crop performance in support of sustainable agriculture. Therefore, the application of 50% organic fertilizer and 50% chemical fertilizer supplemented with a Bacillus-based microbial consortium is recommended as an effective strategy to improve productivity and ecosystem services in newly cultivated lands.

## Figures and Tables

**Table 1:** Experimental treatment application scheme and quantity (kg/ha).

Treatment	Base Fertilizer					Additional Fertilizer
	Organic Fertilizer	Chemical Fertilizer			Microbial Inoculant	Chemical Fertilizer
	N (3.37%)	N (46.4%)	P_2_O_5_ (12%)	K_2_O (52%)		N (46.4%)
CK	0	0	0	0	0	0
FC	0	175	1150	235	0	345
T50	3560	0	0	235	0	260
T50M	3560	0	0	235	45	260

## Data Availability

Data are contained within the article.

## Abbreviations

The following abbreviations are used in the manuscript:

FC	100% Chemical Fertilizer
T50	50% Organic Fertilizer + 50% Chemical Fertilizer
T50M	50% Organic Fertilizer +50% Chemical Fertilizer + Microbial Inoculants
CK	no fertilizer
SOM	Soil Organic Matter
TN	Total Nitrogen
AN	Ammonium Nitrogen
AP	Available Phosphorus
NN	Nitrate Nitrogen
AK	Available Potassium
